# Supplementary material for: Learning curve and surgical outcome of robotic assisted colorectal surgery with ERAS program
Source: Sci Rep. 2022 Nov 29;12:20566. doi: 10.1038/s41598-022-24665-w (PMC9709162; doi:10.1038/s41598-022-24665-w)
Supplement: Supplementary file 1 — Supplementary Information 1. [file 41598_2022_24665_MOESM1_ESM.zip › LSC Raw data-Ñ[▒K/072/P_ñw╝╨░O▒Kñσ.pdf]

臺中榮民總醫院 臺中市臺灣大道四段1650號 病理部 活體組織切片報告 臨床醫學實驗及病理診斷中心 檢驗(查)報告

[redacted]

Pathologic diagnosis:
1. Sigmoid colon, laparoscopic anterior resection --- a. Ulcer. b. Unremarkable lymph node (0/17).
2. IMA root, dissection --- Unremarkable (0/5).
3. Colon, cut end, distal, resection --- Unremarkable.

Ancillary study for diagnosis:
Special stains for PAS (no fungal element seen), Congo red (-) and Masson's trichome (highlight submucosal fibrosis) and IHC stains for AE1/AE3 (highlight epithelial cells) and CMV (-) are done on section C.

Gross description:
The specimen consists of 1) a segment of colon, 16.6 cm in length and 4.8 cm and 4 cm in bilateral cut end circumference, respectively, and mesocolic soft tissue. An ulcer, 2.1x1.2 cm, is noted, 2 cm to the smaller cut end. On cross section, the ulcer involves to the submucosa with 48 mm to the radial margin, and serosal retraction is also noted. The both cut ends are free of tumor grossly. 2) IMA root, a piece of tan yellow soft tissue, 4x1.8x1 cm. 3) distal cut end, a piece of tan mucosa-covered soft tissue, 2.6x2.2x1 cm. Representative part for section: A) proximal cut end B) distal cut end C-D, K-L) ulcer (ulcer all for section) E) radial margin F) all of specimen 2 G) specimen 3 H-J) lymph nodes. Blue ink indicated serosa; yellow ink indicated radial margin.
#T-59300_2 #M-38000_2 0 1105 000000
#T-C4560_2 #M-09183_2 0 1105 000000

已電子簽章

敬告：本單僅供醫師參考使用，不具任何申請效力！！
請病患妥為保管，若不慎遺失需自負個人資料外洩責任！
